# Anti-Influenza A Virus Activity of Rhamnan Sulfate from Green Algae *Monostroma nitidum* in Mice with Normal and Compromised Immunity

**DOI:** 10.3390/md18050254

**Published:** 2020-05-13

**Authors:** Masahiro Terasawa, Kyoko Hayashi, Jung-Bum Lee, Kaoru Nishiura, Koichi Matsuda, Toshimitsu Hayashi, Toshio Kawahara

**Affiliations:** 1 Konan Chemical Manufacturing Co., LTD., 1515 Kitagomizuka, Kusu-cho, Yokkaichi, Mie 510-0103, Japan; nisiura@konanchemical.co.jp (K.N.); matsuda@konanchemical.co.jp (K.M.); 2 Graduate School of Engineering, Chubu University, 1200 Matsumoto-cho, Kasugai, Aichi 487-8501, Japan; kyhayashi@cronos.ocn.ne.jp; 3 Faculty of Pharmaceutical Sciences, University of Toyama, 2630 Sugitani, Toyama, Toyama 930-0194, Japan; lee@pha.u-toyama.ac.jp; 4 College of Life and Health Sciences, Chubu University, 1200 Matsumoto-cho, Kasugai, Aichi 487-8501, Japan; hayashit@skyblue.ocn.ne.jp (T.H.); toshi@isc.chubu.ac.jp (T.K.)

**Keywords:** antiviral, influenza virus, rhamnan sulfate

## Abstract

Influenza viruses cause a significant public health burden each year despite the availability of anti-influenza drugs and vaccines. Therefore, new anti-influenza virus agents are needed. Rhamnan sulfate (RS) is a sulfated polysaccharide derived from the green alga *Monostroma nitidum*. Here, we aimed to demonstrate the antiviral activity of RS, especially against influenza A virus (IFV) infection, in vitro and in vivo. RS showed inhibitory effects on viral proliferation of enveloped viruses in vitro. Evaluation of the anti-IFV activity of RS in vitro showed that it inhibited both virus adsorption and entry steps. The oral administration of RS in IFV-infected immunocompetent and immunocompromised mice suppressed viral proliferation in both mouse types. The oral administration of RS also had stimulatory effects on neutralizing antibody production. Fluorescent analysis showed that RS colocalized with M cells in Peyer’s patches, suggesting that RS bound to the M cells and may be incorporated into the Peyer’s patches, which are essential to intestinal immunity. In summary, RS inhibits influenza virus infection and promotes antibody production, suggesting that RS is a potential candidate for the treatment of influenza virus infections.

## 1. Introduction

Influenza viruses are disease-causing agents belonging to the *Orthomyxoviridae* family. There are three types of influenza viruses: A, B, and C. Influenza A and B viruses cause seasonal flu epidemics almost every year around the world. Influenza A viruses (IFVs) infect a broad range of hosts, including humans, pigs, dogs, horses, and birds, and they are known to evolve into highly pathogenic strains. The emergence of novel IFVs have been known to cause pandemics in recent history, such as the influenza pandemic of 2009, and the potential emergence of a highly pathogenic avian influenza virus strain into a pandemic remains a global health concern [1]. In addition to influenza viruses, coronaviruses, such as the causative agents of severe acute respiratory syndrome (SARS), Middle East respiratory syndrome (MERS), and the new SARS-CoV-2 have also recently emerged as threats to global health, thereby necessitating new control strategies against emerging viral infections.

Antivirals and vaccines are the primary tools for controlling viral diseases. Neuraminidase inhibitors such as oseltamivir and zanamivir are often used for the treatment of influenza virus infections. However, drug-resistant influenza strains have been reported [2,3]. Although vaccines are indispensable to the prevention of influenza, the current vaccine design has several limitations: vaccine production using embryonated chicken eggs takes over 6 months, and manufacturing vaccines against avian influenza viruses such as the H5N1 subtype is often difficult because of its high pathogenicity in chickens. To overcome the disadvantages related to neuraminidase inhibitors and vaccine production, other types of drugs that exhibit both anti-influenza virus activities and different mechanisms of action such as stimulating the immune system or inhibiting viral adsorption are needed. 

Seaweeds have been traditionally consumed by people in East Asia. Recent studies have shown that seaweeds have health-promoting properties that make them suitable as functional foods. Fucoidan, a sulfated polysaccharide from brown seaweed, has been successfully isolated and has been reported to have anti-influenza virus activity [4,5]. The intake of fucoidan has also been shown to increase the production of anti-influenza antibodies [6,7]. Thus, sulfated polysaccharides may be able to both reduce viral replication and increase virus-specific antibody production.

*Monostroma nitidum* (*M. nitidum*) is a green alga that grows in shallow waters along the coasts of Japan. Rhamnan sulfate (RS) is another sulfated polysaccharide found in green seaweed [8], and it has recently been identified as a main component of *M. nitidum* [8,9]. The main repeating unit of RS consists of rhamnose with a sulfate-group substituent that forms long linear chains with branched side chains [10,11,12]. Several in vitro and in vivo studies have reported that RS has anticoagulative [12,13,14,15,16,17], antiviral [10,18,19,20], anti-inflammatory [21], and anti-obesity properties [9]. 

Peyer’s patches are observable at the intestinal epithelium as oval or round lymphoid follicles. They are considered as the immune sensors of the intestine, owing to their ability to transport luminal antigens and bacteria [22]. Peyer’s patches are covered by specialized cells called microfold cells (M cells) that capture antigens from the lumen and present the antigens to antigen-presenting cells. Dendritic cells and macrophages can also directly sample the lumen by extending dendrites through transcellular M cell-specific pores [23]. Although RS has a high molecular weight, it may be incorporated into Peyer’s patches through M cells to stimulate immune activity. However, there is currently no evidence on the incorporation of RS into Peyer’s patches through M cells.

In this study, we first aimed to show the antiviral activities of RS against a broad range of viruses (enveloped and non-enveloped) in vitro. Next, we focused on the characterization of the antiviral effects of RS against IFV infection in vitro and in vivo. In addition, we show histological evidence that RS molecules could bind to M cells in the intestinal epithelium of mice. Our findings suggest that RS is a potential candidate for development into an anti-influenza therapeutic agent.

## 2. Results

### 2.1. Effects of RS on In Vitro Replication of Enveloped and Non-Enveloped Viruses

To assess the antiviral spectrum of RS, we examined its effects on the growth of different host cells and on the replication of different viruses. Representative enveloped viruses (herpes simplex virus 1, HSV-1; HSV-2; human cytomegalovirus, HCMV; measles virus; mumps virus; IFV; human immunodeficiency virus, HIV; and human coronavirus) and non-enveloped viruses (adenovirus, poliovirus, coxsackie virus, and rhinovirus) from different taxonomical groups were used in this study. The half-maximal cell growth inhibitory concentration of RS for each cell type (CC_50_) and the half-maximal effective concentration of RS for each virus (EC_50_) were determined (Table 1). The selectivity indices (SIs) against the viruses were calculated (SI = CC_50_/EC_50_) and are also presented in Table 1. SI values higher than 10 were considered suggestive of antiviral activity. RS showed potent antiviral activities against enveloped viruses (HSV-1, HSV-2, HCMV, measles virus, mump virus, IFV, HIV, and human coronavirus) based on their SIs. On the other hand, RS showed little or no antiviral activity against the non-enveloped viruses (adenovirus, poliovirus, coxsackie virus, and rhinovirus).

The SI values of RS when added during viral infection (Table 1A) were higher than the values obtained when RS was added immediately after infection (Table 1B) for all the enveloped viruses except for HIV. These results suggest that RS has antiviral effects against these enveloped viruses and that RS interferes with the early steps of viral replication, including viral attachment to and penetration into the host cells. In order to characterize the antiviral activity of the RS, its effects on IFV replication were subsequently evaluated.

### 2.2. In Vitro Anti-Influenza Virus Targets

**Figure 1:**
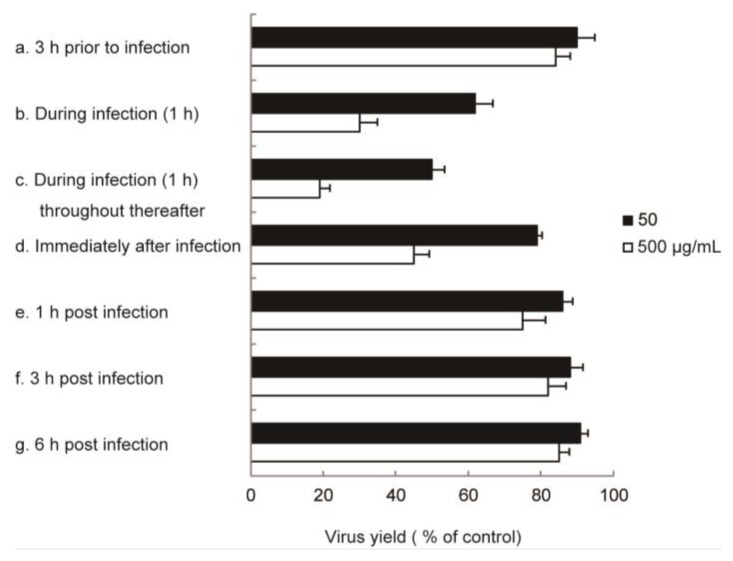
Effects of time-of-addition of RS on IFV replication. RS at a concentration of 50 µg/mL (closed bar) or 500 µg/mL (open bar) was added to the culture medium 3 h prior to virus infection (**a**), during infection for 1 h (**b**), during infection for 1 h, and throughout the subsequent incubation (**c**), immediately after infection (**d**), at 1 h post-infection (p.i.) (**e**), at 3 h p.i. (**f**), or at 6 h p.i. (**g**). Each value is expressed as the mean ± SD from independent duplicate assays.

To clarify the most sensitive step of IFV replication in the presence of RS, time-of-addition experiments were performed. As shown in Figure 1, pretreating host cells for 3 h prior to viral infection did not markedly inhibit IFV replication. In addition, no marked inhibition of viral replication was observed when RS was added during virus infection at 1 h, 3 h, or 6 h post-infection. RS suppressed virus production most efficiently only when added during infection for 1 h and at the same time of infection and throughout the subsequent incubation.

**Figure 2:**
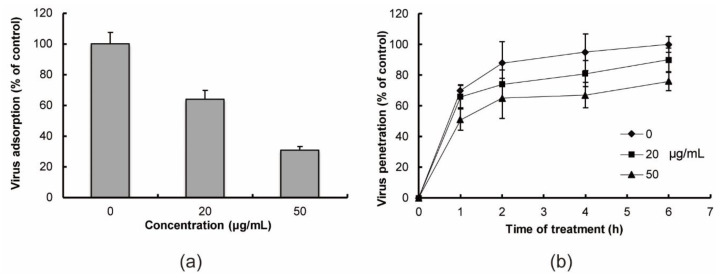
Effects of RS on virus adsorption and penetration. (**a**) For viral adsorption inhibition assays, Madin–Darby canine kidney (MDCK) cell suspensions were infected with IFV (1 PFU/cell) in the absence or presence of RS (20 or 50 μg/mL) at 4 °C for 1 h prior to plaque assays. (**b**) In viral penetration assays, cell monolayers were infected at 4 °C with IFV in the absence of RS, and then shifted to 37 °C to allow virus penetration in the presence of RS for indicated time. Virus titers were determined by the plaque assay. Closed diamond, no drug control; closed square, 20 μg/mL RS; and closed triangle, 50 μg/mL RS. The number of plaques in the control 6 h after temperature shift was assigned as 100%. Each value is the mean ± S.D. from triplicate assays.

These results of time-of-addition experiments suggested that the antiviral targets might be early events including virus adsorption to the host cell surface and/or virus penetration into host cells. Therefore, to evaluate the effects of RS on the early steps of viral replication, we determined whether it could inhibit IFV adsorption and penetration (Figure 2). Treatment with 20 and 50 μg/mL RS markedly inhibited IFV adsorption to Madin–Darby canine kidney (MDCK) cells (Figure 2a). Viral penetration was also inhibited by RS in a dose-dependent manner (Figure 2b), but the effects of RS on viral penetration were lower than its effects on viral adsorption (Figure 2a,b). These results show that RS targets the IFV adsorption and penetration steps of viral replication in vitro.

### 2.3. Effects of RS on Body Weight and Mortality in Immunocompetent and Immunocompromised Mice

**Figure 3:**
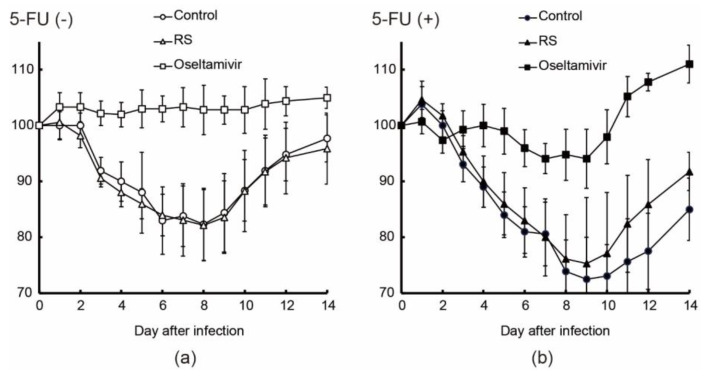
Effects of RS and oseltamivir on mouse body weight change. IFV-infected mice were subcutaneously injected with or without 5-FU, and they were orally administered with water (open and closed circles; control), 5 mg RS (open and closed triangles), or 0.5 mg oseltamivir (open and closed squares) per day. Changes in body weight in 5-FU (-) mice (**a**) and 5-FU (+) mice (**b**) are shown (*n* = 6–11). Bodyweight on the day of viral infection (0 d) was taken as 100%.

To show the effects of RS treatment on immunocompetent and immunocompromised IFV-infected (2 × 10^5^ PFU/50 μL) mice (*n* = 16–21 per treatment group), we induced the immunocompromised state by frequent injections with 5-fluorouracil (5-FU). Among 16–21 mice, we used 6–10 mice to evaluate the effects of RS or oseltamivir on body weight and lethality both on immunocompetent and immunocompromised IFV-infected mice. In the control group (orally administered with water), the immunocompetent mice without 5-FU treatment (5-FU (-) mice) and immunocompromised mice with 5-FU treatment (5-FU (+) mice) showed approximately 20% and 30% reduction in body weight, respectively, 8 days after infection (Figure 3a,b). RS-treated mice with and without 5-FU treatment showed approximately 20% and 25% reduction in body weight, respectively. The 5-FU (-) mice administered with oseltamivir showed no marked weight loss. The oseltamivir-treated 5-FU (+) mice had a 5% reduction of body weight from 7 to 9 days after infection and had recovered by 11 days after infection.

All the 5-FU (-) mice survived throughout the duration of the experiment (Table 2). In the 5-FU (+) mice, one mouse was euthanized and one was found dead in the control group, whereas no mouse death was observed in the RS- and oseltamivir-treated groups (Table 2). These results indicate that RS could protect immunocompetent and immunocompromised mice from lethal IFV infection.

### 2.4. Effects of RS on Virus Replication in Mice

**Figure 4:**
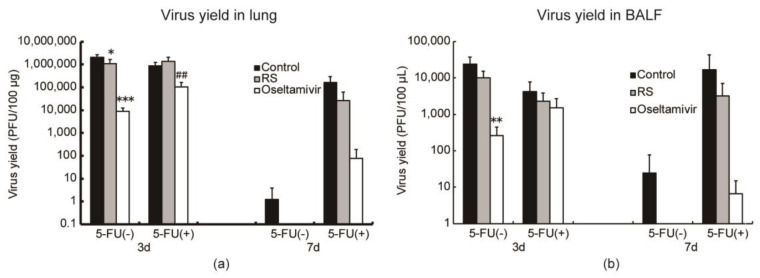
Effects of RS and oseltamivir on virus production in lung and bronchoalveolar lavage fluid (BALF) samples of mice taken 3 or 7 days after IFV infection. IFV-infected mice were subcutaneously injected with or without 5-fluorouracil (5-FU) and were orally administered with water (closed columns, control), 5 mg RS (gray columns), and 0.5 mg oseltamivir (open columns). Virus yields in the (**a**) lung and (**b**) BALF samples are shown. Each value represents the mean ± S.D (*n* = 5). * *p* < 0.05, ** *p* < 0.01, *** *p* < 0.001 versus the control group. ^##^ *p* < 0.01 against the oseltamivir-administered FU (-) group.

To evaluate the effects of RS on viral proliferation, virus yield in the lungs and bronchoalveolar lavage fluids (BALFs) were determined on both 5-FU (-) and 5-FU (+) mice 3 and 7 days after infection. Among 16–21 mice, 5 mice were used to determine the viral yields on each time point. RS significantly reduced IFV production in the lungs to 50% (*p* < 0.05) (Figure 4a and Figure S1a) and showed a tendency to reduce viral titer in the bronchoalveolar lavage fluids (BALFs) of the 5-FU (-) mice 3 days after infection (Figure 4b and Figure S1b). Meanwhile, RS did not reduce lung viral titers (Figure 4a and Figure S1a) but showed a tendency to reduce viral production in the BALFs of the 5-FU (+) mice 3 days after infection (Figure 4b and Figure S1b). No virus was detected in the RS-treated 5-FU (-) mice, but the virus could still be detected in the control mice 7 days after infection (Figure 4 and Figure S1). In the 5-FU (+) mice, RS suppressed viral replication in both lungs and BALFs at 7 days after infection, in contrast with the control group, which had persistent influenza replication (Figure 4 and Figure S1). These results indicate that RS exerted inhibitory effects on IFV replication in both immunocompetent and immunocompromised mice.

### 2.5. Effects of RS on Antibody Responses in Sera

**Figure 5:**
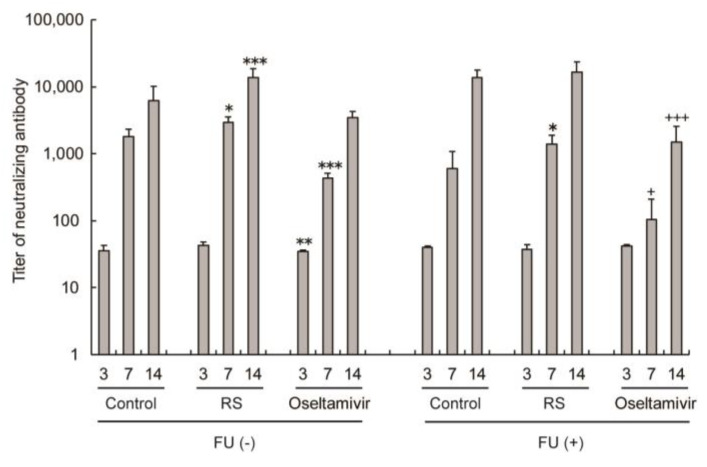
Effects of RS and oseltamivir on the production of neutralizing antibodies against IFV in serum samples of mice taken at 3, 7, or 14 days after IFV infection. IFV-infected mice were subcutaneously injected with or without 5-FU and were orally administered with water (control), 5 mg of RS, and 0.5 of mg oseltamivir. Titers of neutralizing antibody in the serum are shown. Each value represents the mean ± S.D (days 3 and 7, *n* = 5; day 14, *n* = 6–11). * *p* < 0.05, ** *p* < 0.01, *** *p* < 0.001 versus the control group. ^+^ *p* < 0.05, ^+++^ *p* < 0.001 versus the control or RS-administered group.

To evaluate the effects of RS on antibody responses, 5-FU (-) and 5-FU (+) mice were infected with IFV, and sera were collected 3, 7, and 14 days after infection. The effects of RS on the immune response were evaluated in IFV-infected mice at 3, 7, and 14 days after infection. RS induced higher titers of neutralizing antibodies in the sera at 7 and 14 days after infection in both 5-FU (-) and FU (+) mice (Figure 5). On the other hand, oseltamivir significantly suppressed antibody production at 3 and 7 days after infection in FU (-) mice and at 7 and 14 days after infection in FU (+) mice. These results indicate that RS stimulated the production of virus-specific antibodies.

### 2.6. Detection of RS Uptake by M Cells

**Figure 6:**
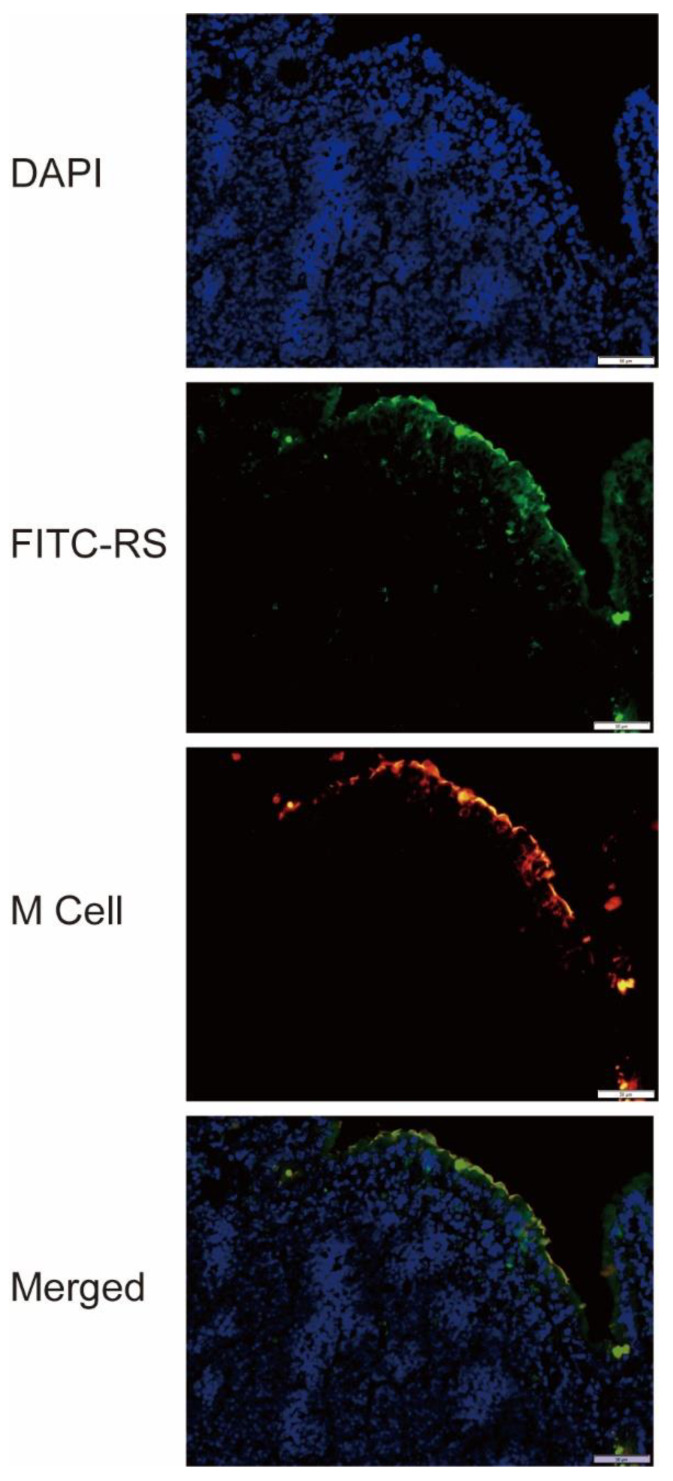
Colocalization of RS and microfold cells (M cells) on the intestinal epithelium of mice. Fluorescein isothiocyanate (FITC)-labeled RS was orally administered to mice, and samples of the intestinal epithelium were collected 30 min after administration. Histological analysis of intestinal tissues stained by FITC-labeled RS, Rhodamine-labeled *Ulex Europaeus* Agglutinin I (M cell), and 4’,6-diamidino-2-phenylindole (DAPI) (nuclei) are shown. Bars: 50 μm.

To clarify the mechanism of RS on reducing virus yield and enhancing antibody production, the localization of RS in the gastrointestinal tract was determined using fluorescein isothiocyanate (FITC)-labeled RS and used for subsequent analysis. FITC-RS was not clearly observed in the sections of Peyer’s patches from the mice at 5 and 60 min after oral administration (data not shown). After 30 min of administration, the fluorescence of FITC-RS was clearly observed in almost all M cells in the Peyer’s patches, which are the immune sensors of the intestine (Figure 6). These data show that RS passed through M cells and entered the Peyer’s patches within 30 min after oral administration. When FITC was orally administered, the substance itself was not detected in M cells after 30 min (data not shown).

## 3. Discussion

In this study, we demonstrated that RS obtained from *Monostroma nitidum* exerted potent inhibitory effects on the proliferation of enveloped viruses including IFV, HSV-1, HSV-2, HCMV, measles virus, mumps virus, HIV, and human coronavirus (Table 1). RS showed high selective toxicity against IFV proliferation based on our calculated SI values. Furthermore, RS exhibited more potent antiviral activity when added before infection than when added after infection. Through in vitro experiments, we have demonstrated that the targets of RS in the IFV replication cycle are adsorption to and penetration into the cells. However, based on the results that show that RS interferes with the early steps of IFV infection, we failed to evaluate the effects of topical administration in vivo. A model to evaluate the effectiveness of treatment and the prevention of IFV infection by the topical administration of RS may be designed to further examine the effects of RS on influenza in the future.

Heparin is a sulfated polysaccharide that is known to bind a glycoprotein, gp120, which is located on the HIV envelope [24,25,26]. Fucoidan also binds gp120 and has anti-HIV activity [27]. RS has also been reported to bind the capsid of enterovirus 71 [18]. The ionic interaction between the sulfated polysaccharide molecule and the viral glycoprotein is believed to be the main mechanism for viral inhibition; the RS derived from *M. nitidum* in this study may also bind viruses through a similar mechanism. We speculate that the molecular target of RS on the influenza virus is hemagglutinin (HA), which is a glycoprotein that is found on the viral envelope and contributes to host cell binding. Analysis of the interaction between RS and HA will have to be performed in future studies to verify this hypothesis.

To analyze the effects of RS on IFV infection in vivo, we performed IFV infection experiments in mice. Our results showed that the oral administration of RS led to an early improvement of influenza symptoms based on the reduction of influenza virus titers in normal mice and the suppression of body weight loss in FU-treated mice. In another animal experiment, the intramuscular injection of RS derived from *M. latissimum* exerted a therapeutic effect on enterovirus replication [18]. However, this study is the first to report the efficacy of orally administered RS against viral infection.

Studies have shown that whereas influenza virus shedding in nasopharyngeal secretions can be controlled within a few days in immunocompetent patients, longer durations of viral shedding have been observed after the onset of symptoms in immunocompromised patients [28,29,30]. Thus, immunocompromised patients have higher risks of acquiring severe influenza virus infection, which also appears to be more difficult to prevent and treat [31]. In this study, the immune-suppressed state of animals was induced by frequent subcutaneous injections of an anticancer drug, 5-fluorouracil [32]. As shown in Figure 3b, progressive weight loss owing to virus infection continued for a longer duration, and the recovery from weight loss was markedly prolonged in the immunocompromised animals as compared to the immunocompetent mice (Figure 3a,b). The severity of influenza associated with immunosuppression was also reflected in the increased virus production in the 5-FU-treated mice 3 days after virus infection (Figure 4). RS treatment resulted in more rapid weight recovery (Figure 3b) and in protection from death of the animals (Table 2).

Since RS is a high molecular weight substance, it is unlikely to be transported to the respiratory mucosa, where influenza virus proliferates in vivo. Oseltamivir suppresses virus proliferation in the body, but it also suppresses virus-specific antibodies, whereas RS suppresses virus growth and promotes the production of virus-specific antibodies. Based on these results, we speculated that orally administered RS has stimulatory effects on the intestinal immune response. Considering that RS is a macromolecule, we focused on the M cells in Peyer’s patches that can incorporate large molecules, including bacteria [33]. Since the Peyer’s patch, a lymph node organ associated with the small intestine, is important for inducing antigen-specific intestinal immune response, RS will have to be transported into the Peyer’s patch through the M cells. The polysaccharide β-glucan is known to be transported by M cells into Peyer’s patches [34], thereby enhancing intestinal immunity and stimulating immunoglobulin A (IgA) production [35]. Fucoidan, a sulfated polysaccharide, is also known to stimulate natural killer cells and Peyer’s patch cells [36]. Fucoidan is known to be absorbed into the body from the intestine, but the detailed route of adsorption is unknown [37]. In this study, as part of elucidating the mode of action of RS in vivo, we investigated the localization of FITC-labeled RS in the mouse intestine. Our results revealed that orally administered RS passed through M cells about 30 min after administration and was incorporated into the Peyer’s patch (Figure 6). To our knowledge, this is the first study that captures the behavior of RS in vivo.

Taken together, we have shown that RS has an antiviral activity against a broad spectrum of enveloped viruses, such as HSV-1, HSV-2, HCMV, measles virus, mump virus, IFV, HIV, and human coronavirus. Although we have not tested on other types of coronaviruses yet, we suppose that RS might also exert an antiviral effect on them through a similar mechanism. Further examination shows that RS inhibits the early steps of influenza virus infection and enhances neutralizing antibody production. RS is also able to protect immunocompetent and immunocompromised mice from lethal influenza challenge. We also demonstrate that RS is incorporated into the Peyer’s patch through the M cells, which possibly accounts for its ability to enhance antibody production. Therefore, RS may be a potential tool for combatting influenza virus infection and other enveloped viruses. Future studies may be conducted to further examine its effects on other enveloped viruses and for other strains of influenza viruses.

## 4. Materials and Methods

### 4.1. Preparation of RS

Crude RS was isolated from the hot water extract of *M. nitidum* as previously described, with slight modifications [3]. First, 4 g of crude extract was dissolved in 300 mL of H_2_O and treated with actinase E (Kaken Pharmaceutical, Tokyo, Japan) at 50 °C for 16 h; then, it was applied to a Cellufine A-200 (JNC, Tokyo, Japan) anion-exchange chromatography column, and it was successively eluted with 7 M urea in 2 M KCl. The fractions that tested positive in the phenol-H_2_SO_4_ method for the determination of sugars were collected, dialyzed, and freeze-dried. The purified RS showed a single peak with a shoulder in front of the main peak (molecular weight: 5.6 × 10^5^) in gel permeation chromatography.

### 4.2. Cells and Viruses

Vero, MDCK, and HeLa cells obtained from Denka Seiken Co., Ltd. (Tokyo, Japan) were grown in minimum essential medium (MEM) supplemented with 5% fetal bovine serum (FBS). MRC-5 cells obtained from the American Type Culture Collection (ATCC) were grown in Dulbecco’s Modified Eagle Medium (DMEM) supplemented with 10% FBS. MT-4 and Molt-4/HTLV-IIIB cells obtained from Tokyo Medical and Dental University were grown in RPMI-1640 medium supplemented with 10% FBS. HSV-1 (HF strain), HSV-2 (UW 268 strain), measles virus (Toyoshima strain), mumps virus (EY strain), poliovirus type 3 (Sabin strain), and coxsackievirus type B-1 (Conn-5 strain) were kindly donated by the Toyama Institute of Health and were grown on Vero cells. Influenza A virus (A/NWS/33, H1N1 subtype) (IFV) obtained from Denka Seiken Co., Ltd. was grown on MDCK cells. Human cytomegalovirus (Towne strain) (HCMV) obtained from Kanazawa University and human coronavirus (229E strain) obtained from the ATCC were grown on MRC-5 cells. Adenovirus (type 2) from Toyama Institute of Health and human rhinovirus type 14 (1059 strain) from Maruishi-Pharm Co. (Osaka, Japan) were propagated on HeLa cells. HIV obtained from Denka Seiken Co., Ltd. was prepared from the culture supernatant of persistently infected Molt-4/HTLV-IIIB cells.

### 4.3. In Vitro Antiviral Assays

For cell growth inhibition studies, cells were cultured for 72 h in the presence of increasing concentrations of RS. Viable cell yields were determined by the trypan blue exclusion test. The inhibition data were plotted as dose–response curves, from which the 50% cell growth inhibitory concentration (CC_50_) was obtained. In the antiviral assays, the plaque yield reduction assay was employed except for human coronavirus, adenovirus, and HIV. Cell monolayers in 48-well plates were infected with the virus at a dose of 0.1 plaque-forming units (PFU) per cell at room temperature. After 1 h of viral infection, the monolayers were incubated at 37 °C. Samples were added during infection and throughout the incubation period or immediately after virus infection. Virus yields were determined by plaque assay after 1 day of incubation for HSV-1, HSV-2, measles virus, mumps virus, poliovirus, coxsackie virus, and IFV, and after 5 days of incubation for HCMV. For anti-human coronavirus and anti-adenovirus assays, cells were infected with the virus at 0.01 TCID_50_ (50% tissue culture infectious dose) per cell and incubated in the medium for 3 days. The TCID_50_ values (titers) of the viruses were determined based on cytopathic effects through the Reed–Muench method [38]. For anti-HIV assays, the virus was collected from the supernatants of Molt-4/HTLV-IIIB cell cultures and its TCID_50_ was calculated in MT-4 cells. Mt-4 cells were infected with HIV at 100 TCID_50_ at room temperature for 1 h and incubated at 37 °C for 5 days to count viable cells. Antiviral activity was expressed as the 50% effective concentration (EC_50_), which was the sample concentration that reduced plaque numbers by 50% in treated cultures compared with no-drug controls. Antiviral activities were estimated by selectivity indices (SIs) calculated from CC_50_ and EC_50_ values (SI = CC_50_/EC_50_).

### 4.4. Time-of-Addition Experiments

MDCK cell monolayers were infected with IFV at 2 PFU/cell for 1 h at room temperature. RS was added to the culture medium at concentrations of 50 and 500 μg/mL 3 h before infection, during infection, at 1 h post-infection (p.i.), at 3 h p.i., or at 6 h p.i. The culture media were harvested at 24 h p.i. and subjected to plaque assay.

### 4.5. Virus Adsorption Assay

IFV (100 PFU/dish) and RS were pre-cooled at 4 °C for 2 h before mixing. The virus and RS were mixed and added immediately to MDCK cell monolayers in 35-mm dishes pre-cooled at 4 °C for 2 h; then, they were incubated at 4 °C for 1 h prior to plaque assay.

### 4.6. Virus Penetration Assay

MDCK cell monolayers pre-cooled at 4 °C for 2 h were infected with IFV at 4 °C for 1 h in the absence of RS. After washing 3 times with ice-cold PBS, the cell monolayers were incubated at 37 °C in the medium containing RS. At 0, 1, 2, 4, and 6 h after a temperature shift to 37 °C, the cell monolayers were treated with a 40 mM citrate buffer (pH 3.0) for 1 min to inactivate uninternalized viruses, and they were then overlaid with the medium for plaque assay.

### 4.7. Animals

Female BALB/c mice (5 weeks old) were obtained from Japan SLC (Shizuoka, Japan). All animal experiments were conducted in accordance with the animal experimentation guidelines of the University of Toyama and of Chubu University and approved by the Animal Care Committees at the University of Toyama (A-2006M-41) and of Chubu University (3010057). All mice with a decrease in body weight greater than 20% during one day were euthanized within 24 h (experimental endpoint) by anesthesia. No observable side effects such as diarrhea owing to drug administration were detected throughout the experiments.

### 4.8. Animal Experiments

Mice (16–21 per treatment) were intranasally infected with 2 × 10^5^ PFU of virus in 50 μL phosphate-buffered saline (PBS) on day 0. RS or oseltamivir was given by oral administration at a dose of 5 mg/day or 0.2 mg/day, respectively, twice a day from 7 days before virus inoculation to 7 days after virus inoculation, starting from 30 min after virus infection. In the immunocompromised groups of mice, 5-FU was injected subcutaneously every other day at a dose of 0.5 mg/treatment starting from 7 days before virus inoculation to 13 days after virus inoculation, whereas the immunocompetent mice were not injected with 5-FU. Blood and lung samples and bronchoalveolar lavage fluids (BALFs) were collected from each group at 3, 7, or 14 days after virus inoculation. Blood samples were centrifuged at 3000 rpm for 10 min, and the sera were stored at −20 °C. Lung samples were sonicated for 10 s after the addition of 1 μL PBS per mg of lung tissue and centrifuged at 3000 rpm for 10 min to collect the supernatants, which were then stored at −80 °C. BALF samples were prepared by four washes with 0.8 mL ice-cold PBS via a tracheal cannula and centrifuged at 1500 rpm for 10 min to collect the supernatants, which were then stored at −80 °C. Virus titers in the lung and BALF samples were determined by plaque assay on MDCK cell monolayers.

### 4.9. Assay for Neutralizing Antibody

Neutralizing anti-IFV antibody titers were determined through the 50% plaque reduction assay. Serum samples at 5- to 78, 125-fold dilutions (0.1 mL) were mixed with approximately 200 PFU of virus (0.1 mL) and incubated at 37 °C for 1 h. Each mixture was added onto MDCK cell monolayers in 35-mm dishes to measure residual virus infectivity by plaque assay. The neutralizing antibody titer was defined as the highest dilution of the serum that reduced the plaque numbers by 50% compared with the control (without serum).

### 4.10. Preparation of FITC-Labeled RS

RS (1 g) was dissolved in 50 mL of dimethyl sulfoxide with a few drops of pyridine (Kanto Chemical, Tokyo, Japan). Fluorescein isothiocyanate (20 mg; TCI, Tokyo, Japan) was added, followed by the addition of dibutyltin dilaurate (200 mg; Kanto Chemical, Tokyo, Japan), and the mixture was heated for 2 h at 95 °C. To remove the free dye, FITC-RS was dialyzed using a size 20 (MWCO: 14,000) dialysis membrane (FujiFilm, Tokyo, Japan) with water and then dried.

### 4.11. Preparation of Sections from Peyer’s Patches

BALB/c mice were fasted for 3 h and then orally administered with FITC-RS or FITC at a dose of 2 mg/mouse or 1 mg/mouse, respectively. Mice were killed at 10, 30, or 60 min after oral administration. The Peyer’s patches were dissected from the mouse small intestines, fixed in 4% formaldehyde, and were frozen in Tissue-Tek optimal cutting temperature compound (Sakura Finetek Japan Co., Ltd., Tokyo, Japan). Cryosections (7 μm) were cut and stained with rhodamine-labeled *Ulex Europaeus* Agglutinin I (Vector Laboratories Inc., Burlingame, CA, USA) for the detection of M cells. Then, the sections were counterstained with 0.5 μg/mL DAPI (Dojindo, Kumamoto, Japan) and mounted with Aqua-Poly/Mount (Polysciences, Inc., Warrington, PA, USA). The stained sections were analyzed by fluorescence microscope (OLYMPUS IX73, Olympus Corporation, Tokyo, Japan).

### 4.12. Statistical Analysis

Comparison between two groups was made using the Student’s *t*-test.

## 5. Conclusions

Here, we report that RS alleviates infection by interfering with virus adsorption/internalization in vitro and by enhancing the antibody response against infection *in vivo*, not at the stage of virus growth inside the infected cells. Therefore, the mode of action of RS may be different from that of currently approved drugs such as oseltamivir. Further, it seems that a resistant virus against RS hardly appears, even after long-term use. Future studies are needed to further characterize the effects of RS on different types of enveloped viruses to support its role as a potential antiviral candidate.

## Figures and Tables

**Table 1:** Antiviral activity of RS. HCMV: human cytomegalovirus measles virus; HIV: human immunodeficiency virus; HSV-1: herpes simplex virus 1; HSV-2: herpes simplex virus 2; IFV: influenza A virus.

Virus	Host cell	Envelope	Cytotoxicity (CC_50,_ μg/mL)	Antiviral activity (EC_50,_ μg/mL)		Selectivity index (SI) (CC_50_/EC_50_)	
				A ^a^	B ^b^	A	B
**HSV-1**	Vero	+	16,000 ± 640	6.5 ± 0.49	31 ± 2.8	2500 ± 93	**520 ± 28**
**HSV-2**	Vero	+	16,000 ± 640	0.93 ± 0.18	4.7 ± 0.35	17,000 ± 2600	**3400 ± 120**
**HCMV**	MRC-5	+	12,000 ± 920	1.1 ± 0.18	67 ± 9.9	11,000 ± 930	**180 ± 13**
**Measles virus**	Vero	+	16,000 ± 6 40	8.3 ± 0.57	4300 ± 450	1900 ± 210	**3.7 ± 0.29**
**Mumps virus**	Vero	+	16,000 ± 640	1.5 ± 0.19	51 ± 6.4	11,000 ± 910	**310 ± 26**
**IFV**	MDCK	+	17,000 ± 2000	41 ± 6.4	310 ± 42	410 ± 16	**55 ± 1.1**
**HIV**	HeLa	+	9900 ± 780	1.2 ± 0.15	1.2 ± 0.11	8300 ± 370	**8300 ± 190**
**Human coronavirus**	MRC-5	+	12,000 ± 1900	0.77 ± 0.17	0.99 ± 0.13	16,000 ± 990	**12,000 ± 350**
**Adenovirus**	HeLa	-	4300 ± 220	480 ± 42	>1000	9.0 ± 0.37	**<4**
**Poliovirus**	Vero	-	16,000 ± 640	>5000	>5000	<3	**<3**
**Coxsackie virus**	Vero	-	16,000 ± 640	2600 ± 170	>5000	6.2 ± 0.14	**<3**
**Rhinovirus**	HeLa	-	13,000 ± 640	530 ± 29	>1000	25 ± 0.14	**<13**

Each value is the mean ± SD from independent duplicate assays. ^a^ Sample was added during viral infection and throughout the subsequent incubation. ^b^ Sample was added immediately after viral infection.

**Table 2:** Effects of RS and oseltamivir on mortality.

Sample	FU-Treat	Survived/Challenged	Mortality	Day of Death
Control	-	11/11	0%	
RS	-	11/11	0%	
Oseltamivir	-	10/10	0%	
Control	+	9/11	18%	9, 10 d
RS	+	10/10	0%	
Oseltamivir	+	6/6	0%	

## Supplementary Materials

The following are available online at https://www.mdpi.com/1660-3397/18/5/254/s1: Figure S1: Effects of RS and oseltamivir on virus production in the lung and BALF samples of mice taken 3 or 7 days after IFV infection (relative values).
