# Supplementary material for: Anti-Influenza A Virus Activity of Rhamnan Sulfate from Green Algae Monostroma nitidum in Mice with Normal and Compromised Immunity
Source: Mar Drugs. 2020 May 13;18(5):254. doi: 10.3390/md18050254 (PMC7281209; doi:10.3390/md18050254)
Supplement: Supplementary file 1 [file marinedrugs-18-00254-s001.pdf]

marine drugs

Supplementary Information:

Anti-Influenza A Virus Activity of Rhamnan Sulfate from Green Algae *Monostroma nitidum* in Mice with Normal and Compromised Immunity

Masahiro Terasawa [1,*], Kyoko Hayashi [2], Jung-Bum Lee [3], Kaoru Nishiura [1], Koichi Matsuda [1], Toshimitsu Hayashi [4] and Toshio Kawahara [4]

[1] Konan Chemical Manufacturing Co., LTD., 1515 Kitagomizuka, Kusu-cho, Yokkaichi, Mie 510-0103, Japan; nisiura@konanchemical.co.jp (K.N.); matsuda@konanchemical.co.jp (K.M.)

[2] Graduate School of Engineering, Chubu University, 1200 Matsumoto-cho, Kasugai, Aichi 487-8501, Japan; kyhayashi@cronos.ocn.ne.jp

[3] Faculty of Pharmaceutical Sciences, University of Toyama, 2630 Sugitani, Toyama, Toyama 930-0194, Japan; lee@pha.u-toyama.ac.jp

[4] College of Life and Health Sciences, Chubu University, 1200 Matsumoto-cho, Kasugai, Aichi 487-8501, Japan; hayashit@skyblue.ocn.ne.jp (T.H.); toshi@isc.chubu.ac.jp (T.K.)

* Correspondence: terasawa@konanchemical.co.jp; Tel.: +81-59-397-2612; Fax: +81-59-397-2608

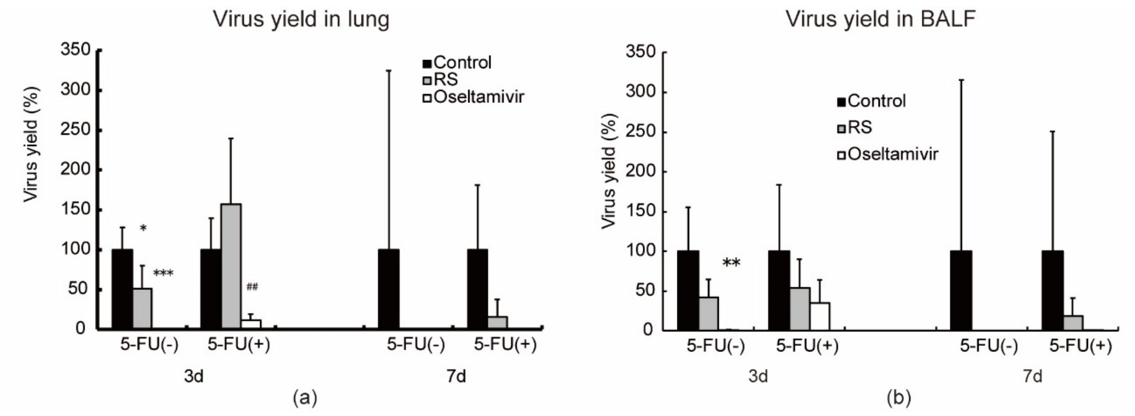

Figure S1. Effects of RS and oseltamivir on virus production in lung and BALF samples of mice taken 3 or 7 days after IFV infection (relative values). IFV-infected mice were subcutaneously injected with or without 5-FU and were orally administered with water (closed columns, control), 5 mg RS (gray columns) and 0.5 mg oseltamivir (open columns). Virus yields in the (**a**) lung and (**b**) BALF samples are shown. The control in each time point was designated to be 100%. Each value represents the mean ± S.D ($n = 5$). * $p < 0.05$, ** $p < 0.01$, *** $p < 0.001$ vs. the control group. ##$p < 0.01$ vs. the oseltamivir-administered FU (-) group.
